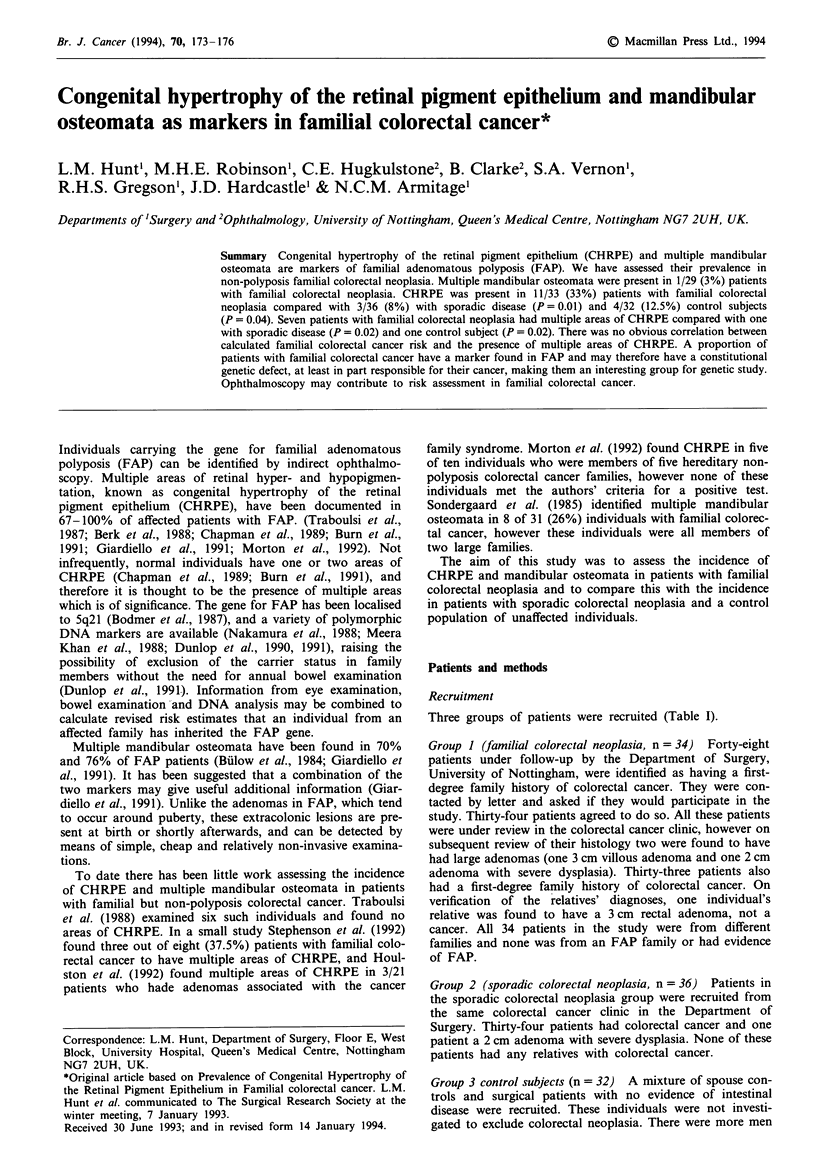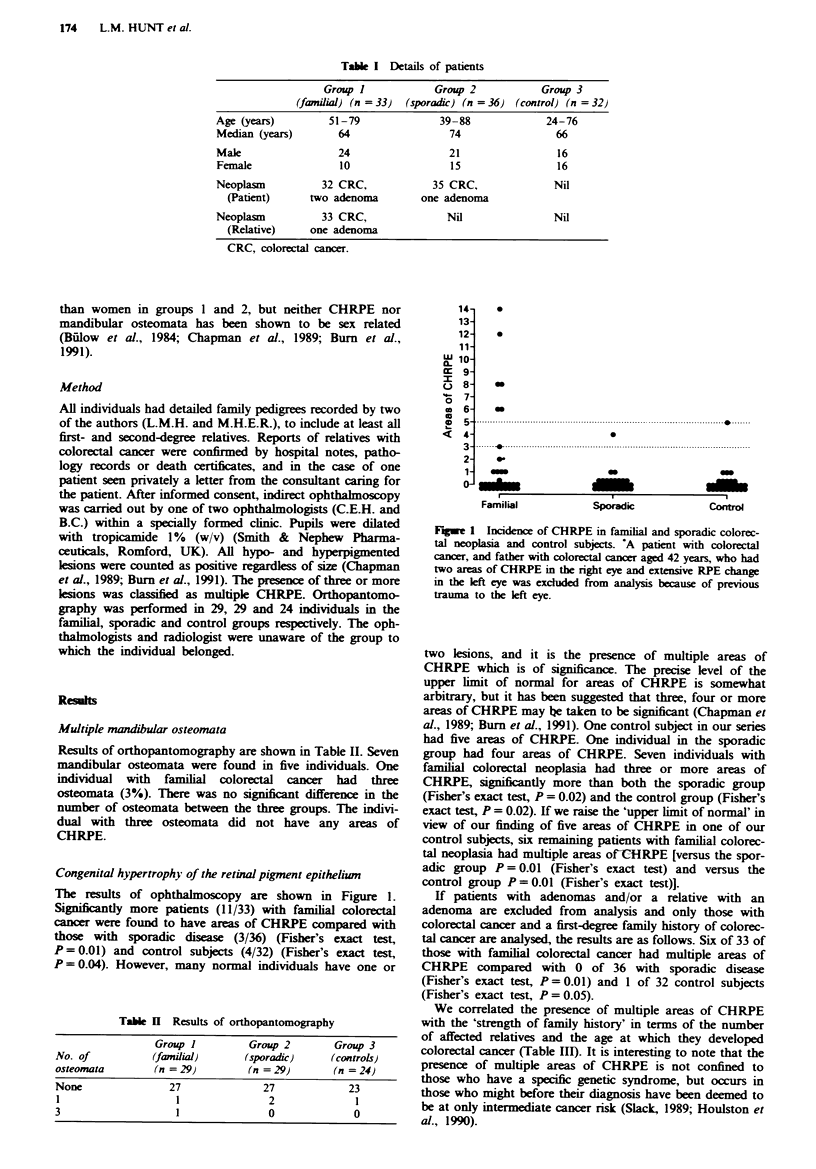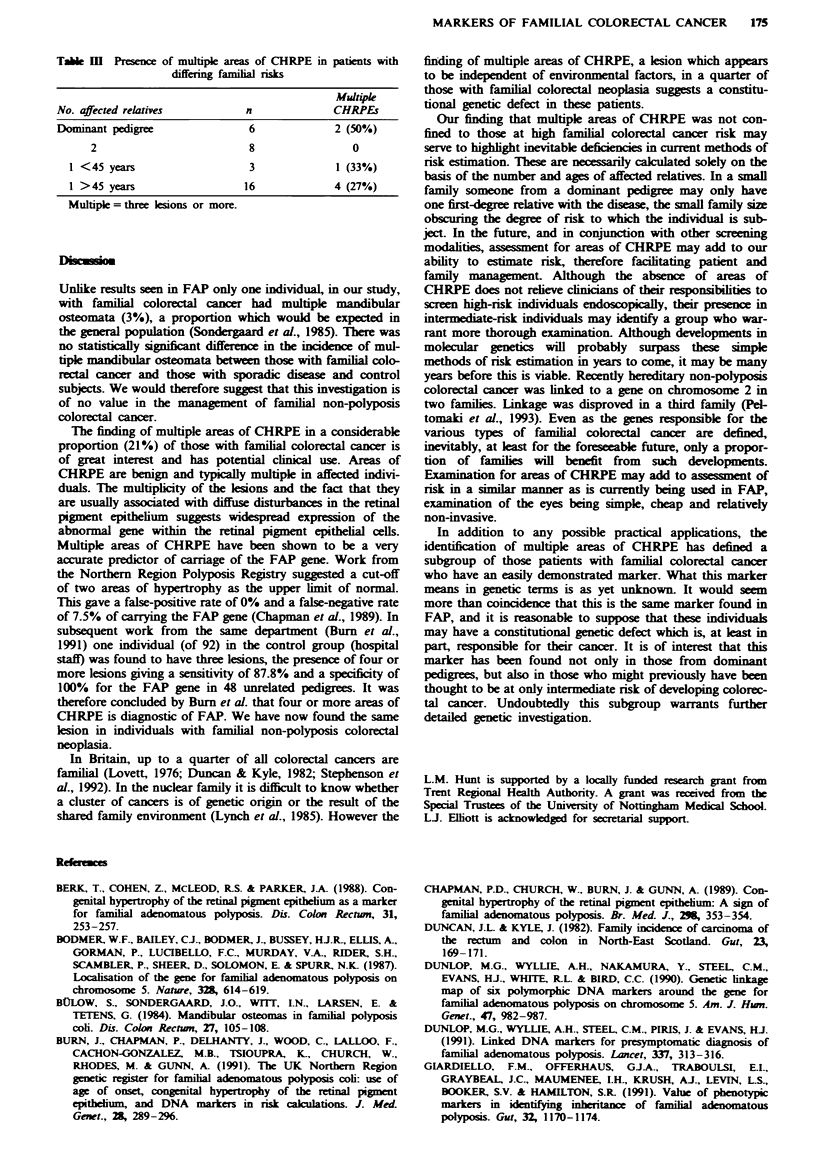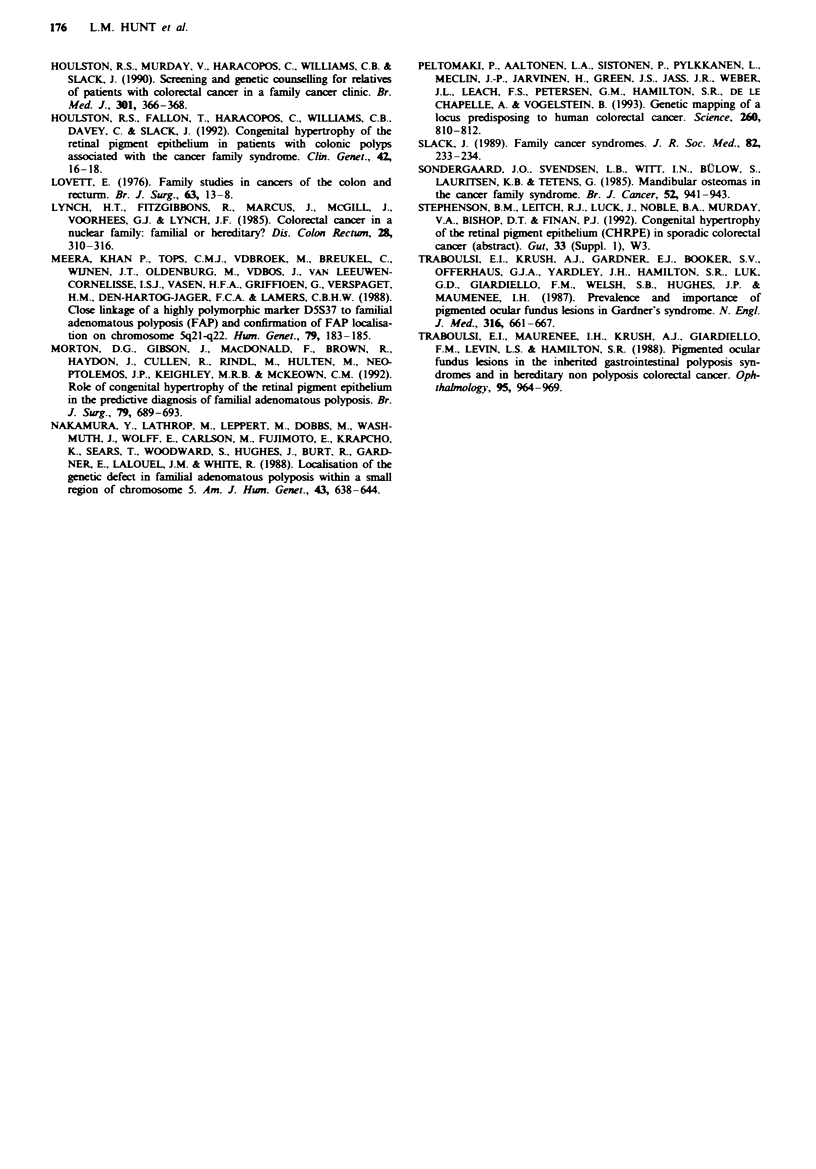# Congenital hypertrophy of the retinal pigment epithelium and mandibular osteomata as markers in familial colorectal cancer.

**DOI:** 10.1038/bjc.1994.271

**Published:** 1994-07

**Authors:** L. M. Hunt, M. H. Robinson, C. E. Hugkulstone, B. Clarke, S. A. Vernon, R. H. Gregson, J. D. Hardcastle, N. C. Armitage

**Affiliations:** Department of Surgery, University of Nottingham, Queen's Medical Centre, UK.

## Abstract

Congenital hypertrophy of the retinal pigment epithelium (CHRPE) and multiple mandibular osteomata are markers of familial adenomatous polyposis (FAP). We have assessed their prevalence in non-polyposis familial colorectal neoplasia. Multiple mandibular osteomata were present in 1/29 (3%) patients with familial colorectal neoplasia. CHRPE was present in 11/33 (33%) patients with familial colorectal neoplasia compared with 3/36 (8%) with sporadic disease (P = 0.01) and 4/32 (12.5%) control subjects (P = 0.04). Seven patients with familial colorectal neoplasia had multiple areas of CHRPE compared with one with sporadic disease (P = 0.02) and one control subject (P = 0.02). There was no obvious correlation between calculated familial colorectal cancer risk and the presence of multiple areas of CHRPE. A proportion of patients with familial colorectal cancer have a marker found in FAP and may therefore have a constitutional genetic defect, at least in part responsible for their cancer, making them an interesting group for genetic study. Ophthalmoscopy may contribute to risk assessment in familial colorectal cancer.


Br. J. Cancer (1994), 70, 173-176 © Macmillan Press Ltd., 1994

### Congenital hypertrophy of the retinal pigment epithelium and mandibular osteomata as markers in familial colorectal cancer*

L.M. Hunt[1], M.H.E. Robinson[1], C.E. Hugkulstone[2], B. Clarke[2], S.A. Vernon[1], R.H.S. Gregson[1], J.D. Hardcastle[1] & N.C.M. Armitage[1]

*Departments of [1]Surgery and [2]Ophthalmology, University of Nottingham, Queen's Medical Centre, Nottingham NG7 2UH, UK.*

**Summary** Congenital hypertrophy of the retinal pigment epithelium (CHRPE) and multiple mandibular osteomata are markers of familial adenomatous polyposis (FAP). We have assessed their prevalence in non-polyposis familial colorectal neoplasia. Multiple mandibular osteomata were present in 1/29 (3%) patients with familial colorectal neoplasia. CHRPE was present in 11/33 (33%) patients with familial colorectal neoplasia compared with 3/36 (8%) with sporadic disease ($P = 0.01$) and 4/32 (12.5%) control subjects ($P = 0.04$). Seven patients with familial colorectal neoplasia had multiple areas of CHRPE compared with one with sporadic disease ($P = 0.02$) and one control subject ($P = 0.02$). There was no obvious correlation between calculated familial colorectal cancer risk and the presence of multiple areas of CHRPE. A proportion of patients with familial colorectal cancer have a marker found in FAP and may therefore have a constitutional genetic defect, at least in part responsible for their cancer, making them an interesting group for genetic study. Ophthalmoscopy may contribute to risk assessment in familial colorectal cancer.

Individuals carrying the gene for familial adenomatous polyposis (FAP) can be identified by indirect ophthalmoscopy. Multiple areas of retinal hyper- and hypopigmentation, known as congenital hypertrophy of the retinal pigment epithelium (CHRPE), have been documented in 67-100% of affected patients with FAP. (Traboulsi *et al.*, 1987; Berk *et al.*, 1988; Chapman *et al.*, 1989; Burn *et al.*, 1991; Giardiello *et al.*, 1991; Morton *et al.*, 1992). Not infrequently, normal individuals have one or two areas of CHRPE (Chapman *et al.*, 1989; Burn *et al.*, 1991), and therefore it is thought to be the presence of multiple areas which is of significance. The gene for FAP has been localised to 5q21 (Bodmer *et al.*, 1987), and a variety of polymorphic DNA markers are available (Nakamura *et al.*, 1988; Meera Khan *et al.*, 1988; Dunlop *et al.*, 1990, 1991), raising the possibility of exclusion of the carrier status in family members without the need for annual bowel examination (Dunlop *et al.*, 1991). Information from eye examination, bowel examination and DNA analysis may be combined to calculate revised risk estimates that an individual from an affected family has inherited the FAP gene.

Multiple mandibular osteomata have been found in 70% and 76% of FAP patients (Bülow *et al.*, 1984; Giardiello *et al.*, 1991). It has been suggested that a combination of the two markers may give useful additional information (Giardiello *et al.*, 1991). Unlike the adenomas in FAP, which tend to occur around puberty, these extracolonic lesions are present at birth or shortly afterwards, and can be detected by means of simple, cheap and relatively non-invasive examinations.

To date there has been little work assessing the incidence of CHRPE and multiple mandibular osteomata in patients with familial but non-polyposis colorectal cancer. Traboulsi *et al.* (1988) examined six such individuals and found no areas of CHRPE. In a small study Stephenson *et al.* (1992) found three out of eight (37.5%) patients with familial colorectal cancer to have multiple areas of CHRPE, and Houlston *et al.* (1992) found multiple areas of CHRPE in 3/21 patients who hade adenomas associated with the cancer family syndrome. Morton *et al.* (1992) found CHRPE in five of ten individuals who were members of five hereditary non-polyposis colorectal cancer families, however none of these individuals met the authors' criteria for a positive test. Sondergaard *et al.* (1985) identified multiple mandibular osteomata in 8 of 31 (26%) individuals with familial colorectal cancer, however these individuals were all members of two large families.

The aim of this study was to assess the incidence of CHRPE and mandibular osteomata in patients with familial colorectal neoplasia and to compare this with the incidence in patients with sporadic colorectal neoplasia and a control population of unaffected individuals.

### Patients and methods

### Recruitment

Three groups of patients were recruited (Table I).

*Group 1 (familial colorectal neoplasia,* n = *34)* Forty-eight patients under follow-up by the Department of Surgery, University of Nottingham, were identified as having a first-degree family history of colorectal cancer. They were contacted by letter and asked if they would participate in the study. Thirty-four patients agreed to do so. All these patients were under review in the colorectal cancer clinic, however on subsequent review of their histology two were found to have had large adenomas (one 3 cm villous adenoma and one 2 cm adenoma with severe dysplasia). Thirty-three patients also had a first-degree family history of colorectal cancer. On verification of the relatives' diagnoses, one individual's relative was found to have a 3 cm rectal adenoma, not a cancer. All 34 patients in the study were from different families and none was from an FAP family or had evidence of FAP.

*Group 2 (sporadic colorectal neoplasia,* n = *36)* Patients in the sporadic colorectal neoplasia group were recruited from the same colorectal cancer clinic in the Department of Surgery. Thirty-four patients had colorectal cancer and one patient a 2 cm adenoma with severe dysplasia. None of these patients had any relatives with colorectal cancer.

*Group 3 control subjects* (n = *32)* A mixture of spouse controls and surgical patients with no evidence of intestinal disease were recruited. These individuals were not investigated to exclude colorectal neoplasia. There were more men

Correspondence: L.M. Hunt, Department of Surgery, Floor E, West Block, University Hospital, Queen's Medical Centre, Nottingham NG7 2UH, UK.
*Original article based on Prevalence of Congenital Hypertrophy of the Retinal Pigment Epithelium in Familial colorectal cancer. L.M. Hunt *et al.* communicated to The Surgical Research Society at the winter meeting, 7 January 1993.
Received 30 June 1993; and in revised form 14 January 1994.

**Table I** Details of patients

| | Group 1 (familial) (n = 33) | Group 2 (sporadic) (n = 36) | Group 3 (control) (n = 32) |
|---|---|---|---|
| Age (years) | 51-79 | 39-88 | 24-76 |
| Median (years) | 64 | 74 | 66 |
| Male | 24 | 21 | 16 |
| Female | 10 | 15 | 16 |
| Neoplasm (Patient) | 32 CRC, two adenoma | 35 CRC, one adenoma | Nil |
| Neoplasm (Relative) | 33 CRC, one adenoma | Nil | Nil |

CRC, colorectal cancer.

than women in groups 1 and 2, but neither CHRPE nor mandibular osteomata has been shown to be sex related (Bülow *et al.*, 1984; Chapman *et al.*, 1989; Burn *et al.*, 1991).

### *Method*

All individuals had detailed family pedigrees recorded by two of the authors (L.M.H. and M.H.E.R.), to include at least all first- and second-degree relatives. Reports of relatives with colorectal cancer were confirmed by hospital notes, pathology records or death certificates, and in the case of one patient seen privately a letter from the consultant caring for the patient. After informed consent, indirect ophthalmoscopy was carried out by one of two ophthalmologists (C.E.H. and B.C.) within a specially formed clinic. Pupils were dilated with tropicamide 1% (w/v) (Smith & Nephew Pharmaceuticals, Romford, UK). All hypo- and hyperpigmented lesions were counted as positive regardless of size (Chapman *et al.*, 1989; Burn *et al.*, 1991). The presence of three or more lesions was classified as multiple CHRPE. Orthopantomography was performed in 29, 29 and 24 individuals in the familial, sporadic and control groups respectively. The ophthalmologists and radiologist were unaware of the group to which the individual belonged.

### Results

### *Multiple mandibular osteomata*

Results of orthopantomography are shown in Table II. Seven mandibular osteomata were found in five individuals. One individual with familial colorectal cancer had three osteomata (3%). There was no significant difference in the number of osteomata between the three groups. The individual with three osteomata did not have any areas of CHRPE.

### *Congenital hypertrophy of the retinal pigment epithelium*

The results of ophthalmoscopy are shown in Figure 1. Significantly more patients (11/33) with familial colorectal cancer were found to have areas of CHRPE compared with those with sporadic disease (3/36) (Fisher's exact test, $P = 0.01$) and control subjects (4/32) (Fisher's exact test, $P = 0.04$). However, many normal individuals have one or

**Table II** Results of orthopantomography

| No. of osteomata | Group 1 (familial) (n = 29) | Group 2 (sporadic) (n = 29) | Group 3 (controls) (n = 24) |
|---|---|---|---|
| None | 27 | 27 | 23 |
| 1 | 1 | 2 | 1 |
| 3 | 1 | 0 | 0 |

**Figure 1** Incidence of CHRPE in familial and sporadic colorectal neoplasia and control subjects. *A patient with colorectal cancer, and father with colorectal cancer aged 42 years, who had two areas of CHRPE in the right eye and extensive RPE change in the left eye was excluded from analysis because of previous trauma to the left eye.

two lesions, and it is the presence of multiple areas of CHRPE which is of significance. The precise level of the upper limit of normal for areas of CHRPE is somewhat arbitrary, but it has been suggested that three, four or more areas of CHRPE may be taken to be significant (Chapman *et al.*, 1989; Burn *et al.*, 1991). One control subject in our series had five areas of CHRPE. One individual in the sporadic group had four areas of CHRPE. Seven individuals with familial colorectal neoplasia had three or more areas of CHRPE, significantly more than both the sporadic group (Fisher's exact test, $P = 0.02$) and the control group (Fisher's exact test, $P = 0.02$). If we raise the 'upper limit of normal' in view of our finding of five areas of CHRPE in one of our control subjects, six remaining patients with familial colorectal neoplasia had multiple areas of CHRPE [versus the sporadic group $P = 0.01$ (Fisher's exact test) and versus the control group $P = 0.01$ (Fisher's exact test)].

If patients with adenomas and/or a relative with an adenoma are excluded from analysis and only those with colorectal cancer and a first-degree family history of colorectal cancer are analysed, the results are as follows. Six of 33 of those with familial colorectal cancer had multiple areas of CHRPE compared with 0 of 36 with sporadic disease (Fisher's exact test, $P = 0.01$) and 1 of 32 control subjects (Fisher's exact test, $P = 0.05$).

We correlated the presence of multiple areas of CHRPE with the 'strength of family history' in terms of the number of affected relatives and the age at which they developed colorectal cancer (Table III). It is interesting to note that the presence of multiple areas of CHRPE is not confined to those who have a specific genetic syndrome, but occurs in those who might before their diagnosis have been deemed to be at only intermediate cancer risk (Slack, 1989; Houlston *et al.*, 1990).

**Table III** Presence of multiple areas of CHRPE in patients with differing familial risks

| *No. affected relatives* | *n* | *Multiple CHRPEs* |
|---|---|---|
| Dominant pedigree | 6 | 2 (50%) |
| 2 | 8 | 0 |
| 1 <45 years | 3 | 1 (33%) |
| 1 >45 years | 16 | 4 (27%) |

Multiple = three lesions or more.

### Discussion

Unlike results seen in FAP only one individual, in our study, with familial colorectal cancer had multiple mandibular osteomata (3%), a proportion which would be expected in the general population (Sondergaard *et al.*, 1985). There was no statistically significant difference in the incidence of multiple mandibular osteomata between those with familial colorectal cancer and those with sporadic disease and control subjects. We would therefore suggest that this investigation is of no value in the management of familial non-polyposis colorectal cancer.

The finding of multiple areas of CHRPE in a considerable proportion (21%) of those with familial colorectal cancer is of great interest and has potential clinical use. Areas of CHRPE are benign and typically multiple in affected individuals. The multiplicity of the lesions and the fact that they are usually associated with diffuse disturbances in the retinal pigment epithelium suggests widespread expression of the abnormal gene within the retinal pigment epithelial cells. Multiple areas of CHRPE have been shown to be a very accurate predictor of carriage of the FAP gene. Work from the Northern Region Polyposis Registry suggested a cut-off of two areas of hypertrophy as the upper limit of normal. This gave a false-positive rate of 0% and a false-negative rate of 7.5% of carrying the FAP gene (Chapman *et al.*, 1989). In subsequent work from the same department (Burn *et al.*, 1991) one individual (of 92) in the control group (hospital staff) was found to have three lesions, the presence of four or more lesions giving a sensitivity of 87.8% and a specificity of 100% for the FAP gene in 48 unrelated pedigrees. It was therefore concluded by Burn *et al.* that four or more areas of CHRPE is diagnostic of FAP. We have now found the same lesion in individuals with familial non-polyposis colorectal neoplasia.

In Britain, up to a quarter of all colorectal cancers are familial (Lovett, 1976; Duncan & Kyle, 1982; Stephenson *et al.*, 1992). In the nuclear family it is difficult to know whether a cluster of cancers is of genetic origin or the result of the shared family environment (Lynch *et al.*, 1985). However the finding of multiple areas of CHRPE, a lesion which appears to be independent of environmental factors, in a quarter of those with familial colorectal neoplasia suggests a constitutional genetic defect in these patients.

Our finding that multiple areas of CHRPE was not confined to those at high familial colorectal cancer risk may serve to highlight inevitable deficiencies in current methods of risk estimation. These are necessarily calculated solely on the basis of the number and ages of affected relatives. In a small family someone from a dominant pedigree may only have one first-degree relative with the disease, the small family size obscuring the degree of risk to which the individual is subject. In the future, and in conjunction with other screening modalities, assessment for areas of CHRPE may add to our ability to estimate risk, therefore facilitating patient and family management. Although the absence of areas of CHRPE does not relieve clinicians of their responsibilities to screen high-risk individuals endoscopically, their presence in intermediate-risk individuals may identify a group who warrant more thorough examination. Although developments in molecular genetics will probably surpass these simple methods of risk estimation in years to come, it may be many years before this is viable. Recently hereditary non-polyposis colorectal cancer was linked to a gene on chromosome 2 in two families. Linkage was disproved in a third family (Peltomaki *et al.*, 1993). Even as the genes responsible for the various types of familial colorectal cancer are defined, inevitably, at least for the foreseeable future, only a proportion of families will benefit from such developments. Examination for areas of CHRPE may add to assessment of risk in a similar manner as is currently being used in FAP, examination of the eyes being simple, cheap and relatively non-invasive.

In addition to any possible practical applications, the identification of multiple areas of CHRPE has defined a subgroup of those patients with familial colorectal cancer who have an easily demonstrated marker. What this marker means in genetic terms is as yet unknown. It would seem more than coincidence that this is the same marker found in FAP, and it is reasonable to suppose that these individuals may have a constitutional genetic defect which is, at least in part, responsible for their cancer. It is of interest that this marker has been found not only in those from dominant pedigrees, but also in those who might previously have been thought to be at only intermediate risk of developing colorectal cancer. Undoubtedly this subgroup warrants further detailed genetic investigation.

L.M. Hunt is supported by a locally funded research grant from Trent Regional Health Authority. A grant was received from the Special Trustees of the University of Nottingham Medical School. L.J. Elliott is acknowledged for secretarial support.

### References

BERK, T., COHEN, Z., McLEOD, R.S. & PARKER, J.A. (1988). Congenital hypertrophy of the retinal pigment epithelium as a marker for familial adenomatous polyposis. *Dis. Colon Rectum*, **31**, 253-257.

BODMER, W.F., BAILEY, C.J., BODMER, J., BUSSEY, H.J.R., ELLIS, A., GORMAN, P., LUCIBELLO, F.C., MURDAY, V.A., RIDER, S.H., SCAMBLER, P., SHEER, D., SOLOMON, E. & SPURR, N.K. (1987). Localisation of the gene for familial adenomatous polyposis on chromosome 5. *Nature*, **328**, 614-619.

BÜLOW, S., SONDERGAARD, J.O., WITT, I.N., LARSEN, E. & TETENS, G. (1984). Mandibular osteomas in familial polyposis coli. *Dis. Colon Rectum*, **27**, 105-108.

BURN, J., CHAPMAN, P., DELHANTY, J., WOOD, C., LALLOO, F., CACHON-GONZALEZ, M.B., TSIOUPRA, K., CHURCH, W., RHODES, M. & GUNN, A. (1991). The UK Northern Region genetic register for familial adenomatous polyposis coli: use of age of onset, congenital hypertrophy of the retinal pigment epithelium, and DNA markers in risk calculations. *J. Med. Genet.*, **28**, 289-296.

CHAPMAN, P.D., CHURCH, W., BURN, J. & GUNN, A. (1989). Congenital hypertrophy of the retinal pigment epithelium: A sign of familial adenomatous polyposis. *Br. Med. J.*, **298**, 353-354.

DUNCAN, J.L. & KYLE, J. (1982). Family incidence of carcinoma of the rectum and colon in North-East Scotland. *Gut*, **23**, 169-171.

DUNLOP, M.G., WYLLIE, A.H., NAKAMURA, Y., STEEL, C.M., EVANS, H.J., WHITE, R.L. & BIRD, C.C. (1990). Genetic linkage map of six polymorphic DNA markers around the gene for familial adenomatous polyposis on chromosome 5. *Am. J. Hum. Genet.*, **47**, 982-987.

DUNLOP, M.G., WYLLIE, A.H., STEEL, C.M., PIRIS, J. & EVANS, H.J. (1991). Linked DNA markers for presymptomatic diagnosis of familial adenomatous polyposis. *Lancet*, **337**, 313-316.

GIARDIELLO, F.M., OFFERHAUS, G.J.A., TRABOULSI, E.I., GRAYBEAL, J.C., MAUMENEE, I.H., KRUSH, A.J., LEVIN, L.S., BOOKER, S.V. & HAMILTON, S.R. (1991). Value of phenotypic markers in identifying inheritance of familial adenomatous polyposis. *Gut*, **32**, 1170-1174.

HOULSTON, R.S., MURDAY, V., HARACOPOS, C., WILLIAMS, C.B. & SLACK, J. (1990). Screening and genetic counselling for relatives of patients with colorectal cancer in a family cancer clinic. *Br. Med. J.*, **301**, 366-368.

HOULSTON, R.S., FALLON, T., HARACOPOS, C., WILLIAMS, C.B., DAVEY, C. & SLACK, J. (1992). Congenital hypertrophy of the retinal pigment epithelium in patients with colonic polyps associated with the cancer family syndrome. *Clin. Genet.*, **42**, 16-18.

LOVETT, E. (1976). Family studies in cancers of the colon and recturm. *Br. J. Surg.*, **63**, 13-8.

LYNCH, H.T., FITZGIBBONS, R., MARCUS, J., MCGILL, J., VOORHEES, G.J. & LYNCH, J.F. (1985). Colorectal cancer in a nuclear family: familial or hereditary? *Dis. Colon Rectum*, **28**, 310-316.

MEERA, KHAN P., TOPS, C.M.J., VDBROEK, M., BREUKEL, C., WIJNEN, J.T., OLDENBURG, M., VDBOS, J., VAN LEEUWEN-CORNELISSE, I.S.J., VASEN, H.F.A., GRIFFIOEN, G., VERSPAGET, H.M., DEN-HARTOG-JAGER, F.C.A. & LAMERS, C.B.H.W. (1988). Close linkage of a highly polymorphic marker D5S37 to familial adenomatous polyposis (FAP) and confirmation of FAP localisation on chromosome 5q21-q22. *Hum. Genet.*, **79**, 183-185.

MORTON, D.G., GIBSON, J., MACDONALD, F., BROWN, R., HAYDON, J., CULLEN, R., RINDL, M., HULTEN, M., NEOPTOLEMOS, J.P., KEIGHLEY, M.R.B. & MCKEOWN, C.M. (1992). Role of congenital hypertrophy of the retinal pigment epithelium in the predictive diagnosis of familial adenomatous polyposis. *Br. J. Surg.*, **79**, 689-693.

NAKAMURA, Y., LATHROP, M., LEPPERT, M., DOBBS, M., WASHMUTH, J., WOLFF, E., CARLSON, M., FUJIMOTO, E., KRAPCHO, K., SEARS, T., WOODWARD, S., HUGHES, J., BURT, R., GARDNER, E., LALOUEL, J.M. & WHITE, R. (1988). Localisation of the genetic defect in familial adenomatous polyposis within a small region of chromosome 5. *Am. J. Hum. Genet.*, **43**, 638-644.

PELTOMAKI, P., AALTONEN, L.A., SISTONEN, P., PYLKKANEN, L., MECLIN, J.-P., JARVINEN, H., GREEN, J.S., JASS, J.R., WEBER, J.L., LEACH, F.S., PETERSEN, G.M., HAMILTON, S.R., DE LE CHAPELLE, A. & VOGELSTEIN, B. (1993). Genetic mapping of a locus predisposing to human colorectal cancer. *Science*, **260**, 810-812.

SLACK, J. (1989). Family cancer syndromes. *J. R. Soc. Med.*, **82**, 233-234.

SONDERGAARD, J.O., SVENDSEN, L.B., WITT, I.N., BÜLOW, S., LAURITSEN, K.B. & TETENS, G. (1985). Mandibular osteomas in the cancer family syndrome. *Br. J. Cancer*, **52**, 941-943.

STEPHENSON, B.M., LEITCH, R.J., LUCK, J., NOBLE, B.A., MURDAY, V.A., BISHOP, D.T. & FINAN, P.J. (1992). Congenital hypertrophy of the retinal pigment epithelium (CHRPE) in sporadic colorectal cancer (abstract). *Gut*, **33** (Suppl. 1), W3.

TRABOULSI, E.I., KRUSH, A.J., GARDNER, E.J., BOOKER, S.V., OFFERHAUS, G.J.A., YARDLEY, J.H., HAMILTON, S.R., LUK, G.D., GIARDIELLO, F.M., WELSH, S.B., HUGHES, J.P. & MAUMENEE, I.H. (1987). Prevalence and importance of pigmented ocular fundus lesions in Gardner's syndrome. *N. Engl. J. Med.*, **316**, 661-667.

TRABOULSI, E.I., MAURENEE, I.H., KRUSH, A.J., GIARDIELLO, F.M., LEVIN, L.S. & HAMILTON, S.R. (1988). Pigmented ocular fundus lesions in the inherited gastrointestinal polyposis syndromes and in hereditary non polyposis colorectal cancer. *Ophthalmology*, **95**, 964-969.